# Does evidentiality support source monitoring and false belief understanding? A cross‐linguistic study with Turkish‐ and English‐speaking children

**DOI:** 10.1111/cdev.13905

**Published:** 2023-03-07

**Authors:** Birsu Kandemirci, Anna Theakston, Ditte Boeg Thomsen, Silke Brandt

**Affiliations:** ^1^ Division of Psychology, Communication, and Human Neuroscience University of Manchester Manchester UK; ^2^ Department of Cross‐Cultural and Regional Studies University of Copenhagen Kobenhavn Denmark; ^3^ Department of Linguistics and English Language Lancaster University Lancaster UK

## Abstract

This study investigates the impact of evidentiality on source monitoring and the impact of source monitoring on false belief understanding (FBU), while controlling for short‐term memory, age, gender, and receptive vocabulary. One hundred (50 girls) monolingual 3‐ and 4‐year‐olds from Turkey and the UK participated in the study in 2019. In Turkish, children's use of direct evidentiality predicted their source monitoring skills, which, in turn, predicted their FBU. In English, FBU was not related to source monitoring. Combined results from both languages revealed that Turkish‐speaking children had better FBU than English‐speaking children, and only for Turkish‐speaking children, better source monitoring skills predicted better FBU. This suggests an indirect impact of evidentiality on FBU by means of source monitoring in Turkish.

**Abbreviations**
BAS II: British Ability Scale, second edition
BPVS‐3: British Picture Vocabulary Scale, third edition
FBU: false belief understanding
ToM: theory of mind

False belief understanding (FBU) is individuals' ability to comprehend that their own and others' beliefs, thoughts, or assumptions might contradict each other and/or reality (e.g., Tomasello, 2018), and it is a defining achievement in young children's cognitive development. FBU acts as a central skill when reasoning about deceiving information and unexpected behavior. There is ample evidence from previous research that cognitive operations such as executive function and memory, as well as children's language skills impact FBU (e.g., Boeg Thomsen et al., 2021; Devine & Hughes, 2014; Wellman et al., 2001). In this study, we focus on the interplay of two closely related under‐investigated factors that might be instrumental to FBU performance: children's development of source monitoring skills and their acquisition of evidentiality marking. Source monitoring refers to the cognitive ability to *track* one's source of information (i.e., the way in which information was gained; e.g., Johnson et al., 1993). Evidentiality, on the other hand, is a linguistic tool that allows (or requires) speakers to *express* their sources of information (e.g., Aikhenvald, 2015). In the following sections, we discuss FBU in more detail and then consider source monitoring and evidentiality as factors that may underpin and support FBU.

## False belief understanding

False belief understanding is considered to be an aspect of a wider cognitive skill, known as theory of mind (ToM), which is children's ability to attribute mental states to themselves and others (Premack & Woodruff, 1978). Earlier studies on FBU suggest that typically developing children master FBU around the ages of 4 to 5 years (e.g., Wimmer & Perner, 1983). However, when age‐appropriate tasks are used and various demands of the tasks such as memory and attention demands are controlled, children at the age of three can pass FBU tasks (Rubio‐Fernández & Geurts, 2013, 2016; see Wellman et al., 2001 for a review). FBU in children is commonly measured by examining their response to an event in which understanding one's own or others' inaccurate beliefs is required. A classic example of such an experimental setting is the *change of location* procedure where the child should appreciate that if an item that a protagonist placed in location X was moved to location Y unbeknownst to them (but clear to the child), the protagonist would continue to believe that the item is in location X (e.g., Baron‐Cohen et al., 1985). When asked to predict where the protagonist will search for the item, children are expected to acknowledge the mismatch between the protagonist's belief and reality, and answer based on the protagonist's point of view (i.e., that they will search in location X). Children's FBU performance has been assessed using implicit measures (e.g., by measuring children's anticipatory looking at the location where the protagonist would search, see Clements & Perner, 1994) as well as explicit measures (e.g., by openly asking children to predict where the protagonist would search, see Wimmer & Perner, 1983). The current study focused on explicit FBU measurement only, due to the debate on non‐replication issues with implicit FBU tests (e.g., Burnside et al., 2018).

False belief understanding can be regarded as a cognitive milestone in a child's development, and several factors have been suggested to contribute to this conceptual development. Language proficiency has been put forward as one of the abilities that support FBU (e.g., Astington & Jenkins, 1999; for a meta‐analysis, see Milligan et al., 2007). However, language proficiency is a multifaceted concept and various aspects of language have been found to play a role in FBU success. For example, it has been suggested that being exposed to mental verbs such as *think* and *know* supports children's false belief development (e.g., Ruffman et al., 2002). Other researchers have suggested that children's proficiency in the syntactical features of the language, such as complement clause understanding, supports the development of FBU (e.g., Boeg Thomsen et al., 2021; de Villiers & Pyers, 2002).

Importantly, the specific way children's linguistic development supports their FBU development depends on the characteristics of the language that the children learn. For mental verbs, for instance, some languages, such as Cantonese, Mandarin (e.g., Liu et al., 2008), Puerto Rican Spanish, and Turkish (Shatz et al., 2003) have explicit terms that highlight whether someone's thought is accurate or not (by having different verbs for “I *thought*” and “I *mistakenly thought*”), which were found to support children's FBU. More importantly for the purposes of this article, in some languages, such as Turkish, the grammar requires speakers to specify their sources of information consistently, known as evidentiality (e.g., Aikhenvald, 2015). In a cross‐linguistic study on 3‐ and 4‐year‐old children's selective trust, Lucas et al. (2013) suggested that having evidential markers in a language may support FBU. They discussed this possibility as Turkish‐speaking children in their study outperformed English‐ and Chinese‐speaking children on FBU. This linguistic concept and its potential impact on FBU are one of the main focuses of this article and we discuss this further in the section on Evidentiality below.

## Source monitoring

Source monitoring is the ability to track one's source of information (Johnson et al., 1993). Information about events and situations can be derived from various sources including perceptual (e.g., seeing, hearing), or inferential (e.g., figuring something out using a clue; Gopnik & Graf, 1988; Johnson et al., 1993). Source monitoring ability has two, somewhat distinct, components. One is the child's immediate ability to recognize and recall where their information stemmed from, and the other is their ability to remember the source after a period of time.

Source monitoring ability is suggested to start developing around the age of 3 years (e.g., Gopnik & Graf, 1988). However, up until the age of 5 years, children were still found to make source monitoring errors (i.e., attributing their information to an inaccurate source, Roberts, 2002) and their ability to store information long term was not found to be fully developed (e.g., Gopnik & Graf, 1988; Ögel, 2007). The shift in performance around the age of 5 years can be related to younger children's poorer cognitive skills in terms of remembering information (e.g., Welch‐Ross, 2000). However, prompting with forced‐choice questions as opposed to more generic “How do you know?” type of questions (e.g., How do you know, did you see it, did you hear it, or did you understand it from a clue?) was also found to scaffold younger children's source monitoring performance (e.g., Gopnik & Graf, 1988; Welch‐Ross, 1995).

Previous research has suggested that there is a connection between source monitoring and FBU abilities, and influences in both directions have been suggested. For example, Welch‐Ross (2000) posited a mental state reasoning account of source monitoring, specifically in the case of suggestibility in children (that is, how likely children are to be influenced by or accept others' ideas about events they have themselves witnessed or heard). She highlighted that, when 3‐ to 5‐year‐old children were told a story and interviewed by a knowledgeable and a naïve interviewer about this story, if children performed well on FBU tasks, then they were less likely to be misled by a naïve interviewer compared to a knowledgeable one (Welch‐Ross, 1999, 2000). However, children who did not pass FBU tests were unable to differentiate between the naïve and knowledgeable interviewers. This suggests that children who are able to understand how knowledge is gained (in this case understanding the naïve interviewer cannot be a reliable source of knowledge, because they do not know the story) can make judgments about the interviewers' level of knowledge by using mental state reasoning. Following Welch‐Ross's account, Bright‐Paul et al. (2008) demonstrated that source monitoring abilities were associated with ToM skills when controlling for chronological and verbal age in 3‐ to 6‐year‐old children. Additionally, first‐person FBU (i.e., understanding one's own false belief) demands the ability to remember one's own previous belief, and then to update it following the revelation of the accurate information. For instance, when shown a familiar candy box, children will initially expect to see candies in this box, whereas after seeing that the box contains pencils, they need to take on board this unexpected revelation that contradicts with their expectation (Perner et al., 1987). In other words, mastering FBU and having a grasp of the source of one's information are related to each other, but due to their correlational nature, the studies investigating the relation are not informative about direction of causality. It has both been suggested that source monitoring supports FBU (Welch‐Ross, 2000) and that FBU supports source monitoring (Lind & Bowler, 2009). Influence may well be bidirectional, with development in either ability supporting development in the other, and since differences in children's linguistic input may affect developments in both, the pathways between FBU and source monitoring may also differ across children growing up with different languages.

## Evidentiality

To talk about the source of information, there is a need for a linguistic means. Some languages provide support for specifying one's knowledge source or even force the speaker to mention the origin of their knowledge (e.g., Aikhenvald, 2015). Turkish is one such language that distinctly marks the source of the speakers' knowledge by obliging them to mention how they acquired the information when talking about past events (i.e., whether they have witnessed, heard of, or inferred it; e.g., Aksu‐Koç et al., 2009). Below, we describe the grammar of evidentiality in Turkish and summarize studies on children's acquisition of these obligatory grammatical markers of knowledge source. We then turn to expressions of evidentiality in a language that does *not* offer speakers grammatical means for marking knowledge source, English, and summarize the few studies of English‐speaking children's acquisition of lexical means for expressing evidentiality.

In Turkish, evidentiality is marked grammatically by verbal suffixes when referring to past events (e.g., Slobin & Aksu‐Koç, 1982). The two earliest acquired evidential markers Turkish‐speaking children correctly produce are the direct evidential marker “*‐di*” (can be *‐dı*, *‐du*, or *‐dü* depending on the vowel harmony rule, and *‐tı*, *‐ti*, *‐tu*, or *‐tü* depending on the consonant harmony rule), and the inferential evidential marker “*‐miş*” (can be *‐mış*, *‐muş*, or *‐müş* depending on the vowel harmony). “*‐di*” is used to report events in the past that were witnessed by the speaker. For instance in the sentence “*Babam eve gel+di*” ([I have witnessed that] “My father came home”), the speaker is reporting that their father came home and that they were there to witness this. However in the example of “*Babam eve gel+miş*” ([It appears that] “My father came home”), the speaker is reporting that they were not there to witness their father's action. The speaker might have heard this information from someone else or inferred it from another event such as seeing their father's car in the garage.

In spontaneous speech, the direct evidential marker appears as the first verb inflection children produce at the early age of one and a half to 2 years, followed by the inferential evidential marker which emerges a few months later (Aksu‐Koç et al., 2009). Aksu‐Koç and colleagues argue that the hearsay function of *‐miş* is more demanding than the inference function on a cognitive level as it requires comprehending somebody else's utterance and taking on board their experience rather than talking about one's own experience. In an experimental study, children's ability to produce sentences with ‐*di* in the appropriate condition (i.e., when directly experiencing an event acted out with toys) appeared at around 3 to 3.5 years of age (Aksu‐Koç, 1988, as cited in Aksu‐Koç et al., 2009). In the same experiment, children used ‐*miş* in the appropriate condition (i.e., when witnessing the beginning and ending of an event, but not the middle section) at around 4 years. This suggests that there is around a year's delay for evidential markers to be produced accurately in experimental studies when compared to naturalistic data. This might be explained by the characteristics of the tasks as they might not create the appropriate setting for the child to produce the accurate evidential marker. In experimental studies, children often watch videos or look at pictures rather than experiencing the situation first‐hand. For instance, Öztürk and Papafragou (2016) presented videos to 5‐ to 7‐year‐old children where the children either watched an event happening or heard/inferred about events that they had not witnessed (e.g., the character in the video was talking about a past event; or there was a product of an action in the video, omitting the process of production). While procedures similar to this are widely used to measure children's production of evidential markers, a more straightforward experimental design that puts the child as the main character of the story could create less cognitive demand. To this end, Ögel (2007) designed two tasks that center around the participant. Details of both tasks by Ögel (2007) are provided in the Methods section.

Additionally, experiments suggest that Turkish‐speaking children's production of evidential markers predates their comprehension, but given the metalinguistic character of the comprehension experiments, it is likely that they underestimate children's understanding of evidentials in their everyday lives. In one study, children were asked to reason about the usage of certain evidential markers (e.g., Aksu‐Koç & Slobin, 1986). While children at the age of 4 years could recognize that indirect speech is a result of not witnessing the event, it is not until around 5–6 years of age that children can accurately justify why one evidential marker should be used over the other, addressing the directness and indirectness aspect of the information in relation to the locutor (Aksu‐Koç et al., 2009). It is simpler for younger children to produce the accurate evidential marker, but gradually more complicated to match the correct speaker with the correct evidential marker, and explicitly discuss about the reason behind this matching by addressing the evidential markers as a cue for their decision.

We turn now to a language with markedly different means for communicating about sources of information: English, where evidentiality is marked syntactically and lexically rather than morphologically (e.g., Rett & Hyams, 2014), and is optional. It can be marked by the use of perception verb similatives (e.g., My dad *looks/seems/sounds like* he is tired; Rett & Hyams, 2014; also referred to as “copy‐raising constructions” or CRCs, Asudeh & Toivonen, 2012). The specification of one's source of knowledge can also be achieved by using mental or perception verbs and complement clauses (e.g., I *know/saw/heard* that my father came home), modal verbs (e.g., My father *must be* home), or adverbs (e.g., *Apparently/Evidently* my father came home). While they differ from the Turkish suffixes in not being obligatory grammatical evidential markers, these lexical expressions and syntactic constructions also provide details about the source of one's knowledge (e.g., Aikhenvald, 2007).

English‐speaking children were found to produce perception‐verb similatives from around 2 years of age, showing no delay compared to children whose language obligates evidentiality (Rett & Hyams, 2014). The asymmetry that is suggested to exist between the production and comprehension of evidential markers in Turkish also applies to English perception‐verb similatives (Winans et al., 2014). Winans and colleagues presented 4‐, 5‐, and 6‐year‐olds and adults with pictures that depicted direct or indirect evidence for certain events (e.g., pictures of a room with (a) Ernie in bed looking sick, and (b) an empty bed with tissues and medicine around it) and sentences with perception‐verb similatives to be matched with these pictures (e.g., “Ernie looks like he got sick”, and “It looks like Ernie got sick”). Children in all ages showed difficulty in differentiating between direct and indirect evidence and matching pictures with the relevant sentences while adults were able to do so. However, the task might not have been ideal for measuring proficiency with syntactical marking of evidence, as neither of the pictures actually present the child with direct evidence of Ernie being sick; in both cases the child has to infer Ernie's sickness from a picture.

## Cross‐linguistic comparisons

As discussed above, in Turkish, speakers are required to specify the source of their information when discussing past events. English, on the other hand, does not enforce such an obligation on the speakers and the linguistic specification of the source of information is optional. According to Slobin's thinking‐for‐speaking approach, language shapes the way we pay attention to certain aspects of events around us and our way of thinking is affected by the language we speak (Slobin, 1996). Therefore, the diverse structures of these two languages might impact the way the speakers of each language perform in cognitive tasks. Specifically, the way evidentiality or source of information is marked in Turkish and English has been put forward as a reason for the differences in children's performances in both source monitoring and FBU tasks. For instance, Aksu‐Koç et al. (2009) put forward the idea that children's ability to correctly use evidential markers in languages where evidential markers are obligatory might support their source monitoring ability. They suggested that Turkish‐speaking children were at an advantage in source monitoring compared to English‐speaking children and that the reason for Turkish‐speaking children's better performance might be their repeated practice with storing the source of their knowledge which is required to use evidential markers accurately. However, Aksu‐Koç et al.'s (2009, based on Ögel, 2007) research retrospectively compared Turkish‐speaking children's source monitoring skills with the results of an existing study conducted with English‐speaking children (Drummey & Newcombe, 2002), which may be problematic given the different conditions under which these two groups of children completed the experiments. Contrastingly, another study by Papafragou et al. (2007) compared English‐ and Korean‐speaking 3‐ and 4‐year‐olds' source monitoring abilities. Korean is an informative language in this sense as it also obligates the use of evidential markers, similar to Turkish. This cross‐linguistic comparison yielded no advantage for Korean‐speaking children over English‐speaking children in terms of their source monitoring performance. It is thus unclear whether acquiring a language with obligatory and grammatical evidential marking leads to advantages in source monitoring skills.

*If* growing up with a language requiring routine attention to sources of information (i.e., a language with obligatory evidential marking) supports children's source monitoring skills, these skills may in turn promote development in FBU abilities. This would be likely given the previously demonstrated relations between source monitoring and FBU, and indeed, Lucas et al. (2013) demonstrated that 3‐ to 4‐year‐old Turkish‐speaking children outperformed their English‐ and Chinese‐speaking counterparts in FBU tasks. The authors proposed, though did not test, that this difference might stem from the difference in the language systems, namely Turkish having a grammatical evidential system and thus providing more opportunities for Turkish‐speaking children to practice making connections between their information and its source.

While it is likely that the acquisition of obligatory grammatical evidentiality supports Turkish‐speaking children's source monitoring skills, and that this, in turn, leads to advantages in FBU, no study has as yet directly examined the relations between evidentiality, source monitoring and FBU ability in children acquiring languages with and without obligatory evidentiality.

## The current study

The current study was designed to test the suggestions from earlier research that Turkish‐speaking children's acquisition of evidentiality would support their source monitoring abilities (Aksu‐Koç et al., 2009; Ögel, 2007), and that they would be at an advantage in terms of their FBU performance (e.g., Lucas et al., 2013) when compared to English‐speaking children. Given proposals in the literature that source monitoring could both support FBU (e.g., Welch‐Ross, 2000) and be supported by FBU (e.g., Lind & Bowler, 2009), and given that these pathways could differ across children acquiring different languages, we investigated both directions of hypothesized influence in both languages. Turkish‐ and English‐speaking children were exposed to the same experimental procedures, and age, receptive language, and short‐term memory performance were controlled for. We hypothesized that; (1) Turkish‐speaking children's evidentiality performance would predict their source monitoring performance, (2) Turkish‐ and English‐speaking children's source monitoring performance would predict their FBU performance, (3) Speaking Turkish would predict better FBU performance, and (4) The impact of source monitoring skills on FBU would be moderated by language, with a tighter association in Turkish‐speaking children.

Cognitive abilities and demographic variables that might impact children's performance such as their receptive language skills, short‐term memory, gender, and age were included as control variables. While our study includes confirmatory analyses when focusing on the FBU and source monitoring performance in both languages, the evidentiality measurements in English‐speaking children present an exploratory effort, given that the specific evidentiality tasks used in this study were originally designed for Turkish‐speaking children and this study is the first time they were used with English‐speaking children (see details in the Materials section). To our knowledge, this is the first cross‐linguistic study to directly compare Turkish‐ and English‐speaking 3‐ and 4‐year‐old children's FBU performance with a specific interest in their source monitoring ability and evidentiality proficiency.

## METHOD

### Participants

Overall, 100 participants (50 girls, *M*_age_ = 50.1 months, range: 36–59 months) took part in this study across two countries: Turkey and the UK. The sample size was decided based on a simulation‐based power analysis. Before data collection, a dataset was generated in R (R Core Team, 2019) where age, gender, language, short‐term memory, source monitoring, and evidentiality were entered as main variables to predict FBU, and subject and item were entered as random variables. The calculations for the simulation data were based on the means and standard deviations from previous studies where the participants were from similar age groups and did similar tasks. We ran the simulation 100 times and reached high power for the main variables (between .91 and .99) for medium effect size (i.e., .5 Cohen's *d*) when 50 participants per language group were included.

#### Turkish‐speaking participants

Fifty participants (26 boys, *M*_age_ = 50.6 months, range: 42–59 months) from three nurseries on the West coast of Turkey took part in the study. All three nurseries were registered as private nurseries which served middle class Turkish families, and they were chosen based on convenience. All the participants were monolingual speakers of Turkish with no known hearing or language problems.

#### English‐speaking participants

Fifty participants (26 girls, *M*_age_ = 49.6 months, range = 41–59 months, excluding one participant at the age of 36 months, who performed comparably) took part in the study. The majority of the children were tested at their schools and 19 children participated in the study in Lancaster University's child laboratory. The neighborhoods where the schools and the laboratory were located were from the 10% to 40% least deprived areas of England based on the statistics published by the Department for Communities and Local Government (Ministry of Housing, Communities, & Local Government, 2019), and were chosen based on convenience. All the participants were monolingual speakers of English with no known hearing or language problems.

### Materials

Participants completed a set of tasks testing their FBU, source monitoring ability, evidentiality proficiency, short‐term memory, and receptive vocabulary. Detailed scripts for all the tasks can be found on the Open Science Framework project (https://osf.io/mks6h/). The FBU tasks all followed the procedures outlined in Boeg Thomsen et al. (2021), which were designed to reduce memory and attention demands, and communicated with the participants using age‐ and task‐appropriate language.

#### Tests of false‐belief understanding

##### Unexpected identity task

This task was designed building on the Gopnik and Astington (1988) task. The participants were presented with a deceptive pen that looked like a flower and its true identity was later revealed. The experimenter acknowledged the deceiving appearance of the pen. Participants' self (i.e., first person) as well as other (i.e., third person) FBU was measured with this task.

##### Change of location task

This task was designed based on the Wimmer and Perner (1983) and Baron‐Cohen et al. (1985) studies. The experimenter acted out a scenario with toys about an object changing location (a carrot being moved by the protagonist's father from an opaque bag into a pan with a lid) without the protagonist witnessing this change. Participants were only asked a third person FBU question.

##### Unexpected contents task

This task was designed based on the tasks in the Perner et al. (1987) and Hogrefe et al. (1986) studies. The participants were presented with a closed crayons box that had a spoon inside instead of crayons, which was revealed accompanied by a surprise reaction from the experimenter to highlight the unexpectedness of it to the participants. They were asked both first person and third person false‐belief questions. This time the third person false‐belief question was asked first for counterbalancing reasons.

###### Coding and scoring tests of false‐belief understanding

The participants completed these three tasks to receive an overall FBU score. Two of the tasks, Unexpected Identity and Unexpected Contents, measured children's ability to attribute false belief both to themselves and to a third person. For the Change of Location task, there was only a question about third person FBU. As a result, each participant could score between 0 and 5 as their overall FBU score; 2 points each for the Unexpected Contents and the Unexpected Identity tasks, and 1 point for the Change of Location task. Importantly, if the participant responded incorrectly to one of the control questions, then their score for the relevant FBU task would be considered missing, and as a result, their overall FBU score would also be considered missing. Their scores therefore were coded in two ways: individually for each FBU question, and as an overall score for their FBU performance. For instance, if a participant failed to answer the control question for Unexpected Contents correctly, but successfully passed the other control questions for the Unexpected Identity and Change of Location tasks, and additionally answered the false belief questions correctly, they would receive 1 point for the Unexpected Identity self false belief question, 1 point for Unexpected Identity other false belief question, 1 point for Change of Location question, and a missing point for each of the two Unexpected Contents questions. Their overall score would also be missing (NA) as it would be misleading to score 0 points for failing the control question. Zero points would instead be scored if the participant passed the control question(s) but gave an incorrect response to the false belief question.

#### Source monitoring

This task was designed based on Gopnik and Graf (1988) and was referred to as the Mode of Knowledge Access task in Ögel (2007). In the original study, there were three parts to the task: the familiarization part, the immediate recall part, and the delayed recall part. In the current study, we only used the familiarization and the immediate recall parts, given that delayed recall is not established before the age of 5 (Gopnik & Graf, 1988; Ögel, 2007). In the familiarization part, the participants were presented with three colorful envelopes one at a time and were told that there was something hidden in each envelope. The participants were given information about the contents of the envelopes in one of three different ways in each trial: by *seeing* inside the envelope, *hearing* what was in the closed envelope, or *inferring* the contents of the closed envelope from a clue. The participants were then asked what was in each envelope and how they knew it, i.e., the source of their knowledge. They were immediately given the three options: “How do you know that there is X in the envelope? Did you see it, did I tell you, or did you understand it from a clue?”. The participants received feedback regarding their answers, i.e., they got praise for their correct answer(s) and correction and explanation for their incorrect answer(s).

Following the familiarization phase, the participants were presented with the immediate recall part. In this part, they were presented with six colorful opaque paper bags successively, with different items in each one. The procedure for the immediate recall part was mostly the same as the familiarization part, except during the immediate recall part children were not immediately given the three forced‐choice options. If they answered correctly without the forced‐choice questions, the experimenter moved onto the next bag, but if they answered incorrectly, or failed to answer, then the experimenter presented the three options in counterbalanced order.

##### Coding and scoring of the source monitoring task

If the child declared the source of their information correctly, prior to being presented with the options, then they scored 2 points for the relevant item, if they answered correctly after hearing the options, then they scored 1 point, and if they failed to answer correctly (either an incorrect answer or no answer), they scored 0 points. Their score was then calculated as proportion (i.e., the proportion of their score out of 12 possible points).

#### Evidentiality

##### Direct experience evidentiality task

In this task, also known as the Taking a Bath Scenario task, the aim was to capture Turkish‐speaking children's ability to naturally use the direct experience evidential marker (*‐di*) when referring to a past event that they witnessed (Aksu‐Koç et al., 2009; Ögel, 2007). The participants were asked to watch carefully while the experimenter acted out a story using some toys without uttering any words. At the end of the demonstrated story, the experimenter asked the child, “Can you tell me this story?”. The experimenter abstained from using the direct experience marker in the question to avoid influencing the participants' responses.

###### Transcribing and coding of direct evidentiality test

Participants' stories were video‐recorded and transcribed by the experimenter. Twenty percent of the stories produced by the Turkish‐speaking children were also transcribed by an independent native speaker of Turkish for reliability. There was 91% agreement between transcribers and where discrepancies occurred, they were discussed and resolved. Direct experience score was the proportion of the ‐*di* usage in the story as a function of all the verbs that were used. For instance, if a participant told a story where they used six verbs and three of them were inflected with the ‐*di* marker, this participant's score was 50%.

This task was used as an exploratory task for English‐speaking children. As discussed earlier, for English‐speaking children there are lexical and syntactic means to highlight direct evidentiality, such as using complement clauses with perception verbs or using adverbs. English‐speaking children's performances were transcribed and checked for structures such as “I saw that the baby took a bath” or “Evidently the baby took a bath”.

##### Inferential evidentiality task

This task, also known as the Changed State of Objects Task, was implemented following the Ögel (2007) study. The aim of this task was to create a natural context for the Turkish‐speaking participants to use the ‐*miş* inflection when referring to a past non‐witnessed event. The task consisted of two parts, the familiarization part and the actual task. In the familiarization part, the experimenter brought out a toy bag and presented 17 toys. The aim of this first part was to familiarize the child with all the toys and allow them to play freely. Once the participant had some time to familiarize themselves with the toys, the experimenter declared that they had to stop playing as someone else needed the toys, and that they could carry on playing once the toys were back. The participant was asked to engage in another task while waiting. Once they finished this task, the experimenter brought the toys back. While the participant believed that the experimenter brought back the same bag, instead, the experimenter brought a duplicate bag which contained the duplicates of the toys, however, some of the toys were altered (e.g., broken, painted on, or ripped) and one new toy was added. The participant was expected to comment on the changes to the toys by using the ‐*miş* inflection on the verbs (e.g., “arabanın tekerleği çık*‐mış*” [the wheel of the toy car came off]). The experimenter directed the child's attention to the changes to encourage the participant to talk about them when needed, however, abstained from using the evidential marker in their questions.

###### Transcribing and coding of inferential evidentiality task

The task was video recorded, and the relevant parts of the task (i.e., when the participants commented on the changed state of the toys with or without using an inflected verb) were transcribed by the experimenter and an independent coder. Turkish participants' scores for this task were calculated as a proportion of *‐miş* inflected verbs as a function of all the noticed changes to the toys. For instance, if the child recognized five changes overall, and used the *‐miş* inflection when explaining three of them, their score for the task would be 60%.

This task was also used as an exploratory task for English‐speaking children. As mentioned previously, the English language does not require speakers to specify the source of their information when talking about non‐witnessed past events, however, there are options such as mental and modal verbs or adverbs available to them. We were interested in how likely the English‐speaking participants were to use these lexical items. For instance, we recorded the instances where the participants addressed the changes to the toys such as “I *think* the wheel got broken”.

#### Vocabulary

##### British Picture Vocabulary Scale, third edition

The British Picture Vocabulary Scale, third edition (BPVS‐3; Dunn et al., 2009) was used to measure English‐speaking children's receptive vocabulary skills. In this task, the participants were presented with sets of four pictures and were asked to identify the picture that matched the word that the experimenter uttered. The participants continued pointing at pictures until they made 8 or more mistakes within a group of 12 picture sets. The overall number of correct answers was recorded as participants' vocabulary score.

##### Peabody Turkish

The Peabody test for Turkish (Katz, 1974) was used to measure Turkish‐speaking children's receptive vocabulary skills. Similar to the BPVS‐3, the task consists of sets of four pictures and the participants needed to point to the picture that matched the word the experimenter said. Participants continued pointing at pictures until they made eight mistakes overall. The overall number of correct answers was recorded as participants' vocabulary score.

#### Short‐term memory

##### Forward digit span

The Forward Digit Span test from the British Ability Scale, second edition (BAS II; Elliot et al., 2004) was used to measure the participants' short‐term memory. Children heard number sequences (five sequences in each set) and were asked to repeat them. The sequences gradually got longer and two mistakes in a set indicated the termination of the test. For the purposes of this study, the number of correct repetitions represented the child's short‐term memory score.

### Procedure

The study received ethical approval from the Faculty of Arts and Social Sciences and Lancaster Management School's Research Ethics Committee at Lancaster University (UREC: FL16283). Data collection took place in April–November 2019. The parents/caregivers were sent a consent form and the participants were asked for verbal assent. The participants completed all eight tasks in one session that lasted around 25–35 min. The experimenter worked with the participants individually in a quiet part of their nursery/primary school. The order of the tasks was the same for each participant: Unexpected Identity, Change of Location, Source Monitoring Task, Direct Experience Evidentiality Task (Taking a Bath Scenario), Unexpected Contents, Forward Digit Span, Inferential Evidentiality Task (Changed State of Objects)‐familiarization part, BPVS (or Peabody), and Inferential Evidentiality Task (Changed State of Objects)‐actual test.

The session was video recorded for transcribing and coding purposes except for the vocabulary and short‐term memory tasks as these could be coded in real time. Participants received a sticker after each task. The participating schools received book vouchers.

## RESULTS

The results are presented in three parts. First, the results from the Turkish‐speaking participants are presented (referred to as Turkish dataset from here on). Second, the results from the English‐speaking participants are presented (referred to as English dataset from here on). Finally, the results from the combined dataset are presented which comprises data points from both Turkish and English datasets (referred to as Combined dataset from here on).

### Results from the Turkish dataset

First, we focus on the Turkish dataset to address hypothesis 1 (Turkish‐speaking children's evidential marker production performance would predict their source monitoring performance) and the Turkish part of hypothesis 2 (Turkish‐speaking children's source monitoring performance would predict their FBU performance). We begin by presenting descriptive statistics and correlations between the predictor and control variables, then go on to discuss children's evidentiality performance in detail, and finally evaluate predictors of Turkish‐speaking children's source monitoring and FBU performance.

#### Descriptive statistics and correlations

Table 1 summarizes the descriptive statistics for the Turkish dataset. There were 13 missing data points. For the Source Monitoring task, the inferential evidentiality task, and the direct evidentiality task, missing data points reflected the participants' unwillingness to complete the tasks. In the case of the FBU tasks, however, the missing data points were due to the participants' inability to pass the control question(s) for the individual task(s). No participant failed to answer the control questions for all three FBU tasks. The missing data points shown in Table 1 represent the overall number of individual invalid responses for specific tasks.

**TABLE 1:** Descriptive statistics for the Turkish dataset.

	*M*	SD	Range	Max. score	Missing data points (*N* and %)
Short‐term memory	11.38	4.01	0–20	36	0
Receptive vocabulary	48.3	10.59	29–70	100	0
Source monitoring	57.31	28.81	8.33–100	100%	1 of 50 (2%)
False belief	2.74	1.51	0–5	5	10 of 250 (4%)
Evidentiality (direct)	70.71	44.05	0–100	100%	1 of 50 (2%)
Evidentiality (inferential)	64.05	25.2	0–100	100%	1 of 50 (2%)

Table 2 demonstrates the correlations between the continuous predictor and control variables using Spearman's correlation. FBU performance (i.e., collated version of the outcome variable) significantly correlated with source monitoring ability (*r*(48) = .53, *p* < .001) but not with performance with any of the evidentiality tasks. In terms of the control variables, false belief performance significantly and positively correlated with age (*r*_s_(48) = .39, *p* < .001) and short‐term memory (*r*(48) = .38, *p* < .05). Source monitoring performance significantly correlated with direct evidentiality performance, *r*(48) = .34, *p* < .05, but not with inferential evidentiality performance. The control variables (age, vocabulary, and short‐term memory) all correlated with source monitoring performance (all *p*s < .01). Inferential evidentiality performance did not correlate with any of the variables.

**TABLE 2:** Correlations between the predictor and control variables.

Variable	1	2	3	4	5	6	7
1. Age	1						
2. Vocabulary	.50***	1					
3. Short‐term memory	.60***	.49***	1				
4. Source monitoring	.45**	.60***	.48***	1			
5. Evidentiality (direct)	−.02	.26	.19	.34*	1		
6. Evidentiality (inferential)	−.07	.13	.15	.00	−.15	1	
7. False belief	.39**	.15	.38*	.53***	−.13	−.05	1

* *p* < .05

** *p* < .01

*** *p* < .001.

#### Evidentiality performance

**FIGURE 1:**
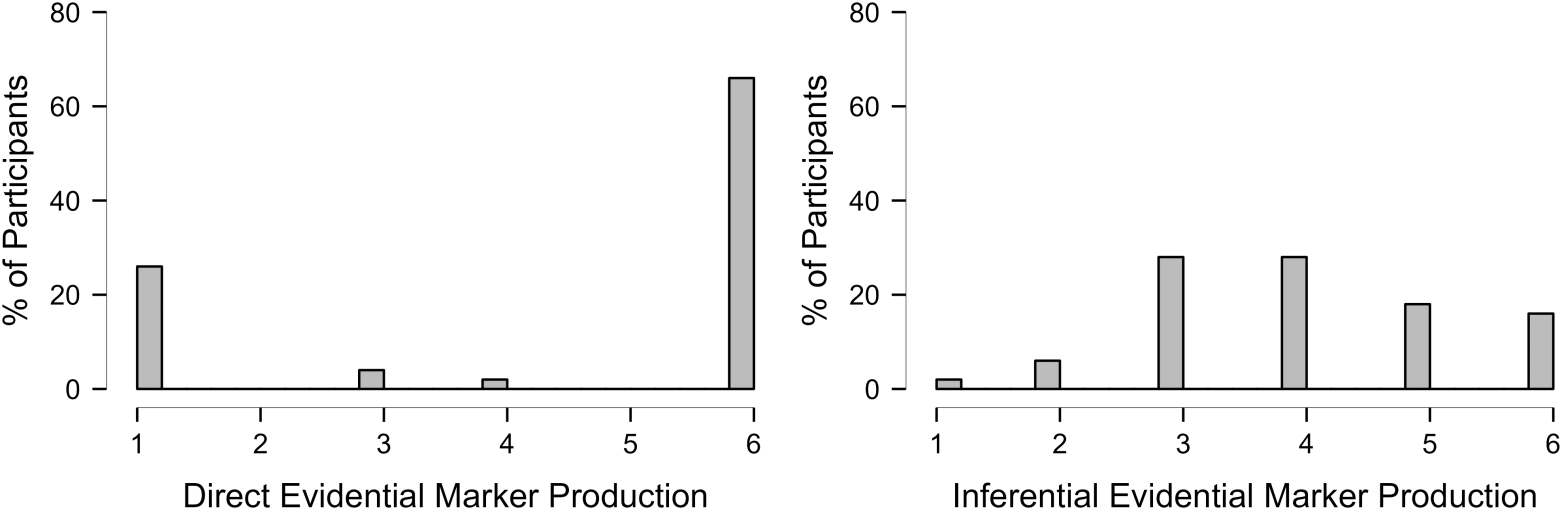
Bar graphs for Turkish‐speaking children's evidentiality performance. Bins on the x axis: 1 = 0%, 2 = 1%–25%, 3 = 26%–50%, 4 = 51%–75%, 5 = 76%–99%, 6 = 100%. Please note that bins 1 and 6 only refer to 0% and 100%, respectively, and are different from the rest of the bins which cover a range. This is to clearly demonstrate the percentage of participants who produced no accurate evidential marker (0%) and produced the accurate evidential marker every time they produced an evidential marker (100%), respectively.

To better understand Turkish‐speaking children's evidentiality performance beyond its contribution to their source monitoring and FBU performance, we further investigate these results. In the direct evidentiality task, 66% of Turkish‐speaking children used the accurate *‐di* inflection every time they used a verb in their story. Twenty‐six percent of the participants did not use *‐di* inflection at all, 6% alternated between *‐di* inflection and a different tense or evidential marker, and a further 2% did not complete this task. Apart from the *‐di* inflection, children used the *‐iyor* inflection which describes an ongoing event and *‐ir* which is used to refer to habitual and/or possible states of affairs, neither of which are evidential markers. For the inferential evidentiality task, 16% of the children used the accurate *‐miş* inflection every time they talked about the changed state of a toy, 2% did not use *‐miş* inflection at all, 80% used it in varying levels, and 2% did not complete this task (see Figure 1 for details). Children who used *‐miş* in varying levels displayed one or more of the following styles: alternated between *‐miş* and *‐di* when talking about the unwitnessed changed state of the object, did not talk about the reason for the change for some of the objects while noticing the change (demonstrated by a sharp inhale or vocal and facial expressions), or used an adjective instead of a verb to describe the change.

#### Predictors of source monitoring performance

To address hypothesis 1 (Turkish‐speaking children's evidentiality performance would predict their source monitoring performance), a multiple linear regression analysis was conducted in R (R Core Team, 2019). The initial model took Source Monitoring as its dependent variable and included all the control and explanatory variables. We then followed the principle of backwards selection (Gries, 2013) where step by step the least significant contributor to the model was excluded to create a new model and an analysis of variance was used to compare the existing model and the new model. If the exclusion of the least significant contributor did not significantly decrease the goodness of fit of the model, the variable was discarded. The final model with all the significant contributors is presented in Table 3.

**TABLE 3:** Multiple linear regression model for source monitoring performance in the Turkish dataset.

Residuals				
Min	1Q	Median	3Q	Max
−41.330	−11.642	4.301	12.753	43.512

Coefficients				
	Estimate	SE	*t* value	Pr(>|*t*|)
(Intercept)	−42.37873	18.26473	−2.320	.025795*
Vocabulary	1.28404	0.33704	3.810	.000494***
Evidentiality (direct)	0.15834	0.07718	2.052	.047142*
False belief understanding	9.41537	2.12158	4.438	.000076***

*Note*: Significance codes: ‘***’ .001, ‘*’ .05.

False belief understanding was the most significant predictor of source monitoring performance, followed by receptive vocabulary performance and direct evidentiality performance. Inferential evidentiality performance did not contribute to source monitoring performance significantly.

#### Predictors of false belief performance

A generalized linear mixed‐effects model analysis using the lme4 package (Bates et al., 2015) in R (R Core Team, 2021) was conducted to address the Turkish part of hypothesis 2 (Turkish‐speaking children's source monitoring performance would predict their FBU performance). The initial model included all the control and explanatory variables and random effects of participant and item. We then followed the same principle of backwards selection as before (Gries, 2013). The final model with all the significant contributors is presented in Table 4.

**TABLE 4:** Generalized linear mixed‐effects model for false belief performance in the Turkish dataset.

Random effects			
Groups	Name	Variance	SD
ID	(Intercept)	.17862	.4226
Item	(Intercept)	.09856	.3139

Fixed effects				
	Estimate	SE	*z* value	Pr(>|*z*|)
(Intercept)	−0.5281	0.2720	−1.942	.052197.
Gender (girls)	1.2211	0.3387	3.606	.000312***
Age	0.4015	0.1849	2.172	.029852*
Source monitoring	0.5607	0.1900	2.951	.003168**

*Note*: Number of observations: 237, groups: ID, 49; Item, 5. Significance codes: ‘***’ .001, ‘**’ .01, ‘*’ .05, ‘.’ .1.

Gender, more specifically, being a girl, was the most significant predictor of FBU performance for Turkish‐speaking participants. Additionally, children's source monitoring abilities and their age were also significant predictors of their FBU performance, but their performance with evidentials was not.

### Results from the English dataset

In this section, we focus on the English dataset to address the English part of hypothesis 2. We begin by presenting descriptive statistics and correlations between the predictor and control variables, then go on to discuss the linguistic tools English‐speaking children used to mark evidentiality, and finally evaluate predictors of English‐speaking children's source monitoring and FBU performance.

#### Descriptive statistics and correlations

Table 5 summarizes the descriptive statistics for the English dataset. Overall, there were 14 missing data points for the English dataset. Two of these were a result of two participants not participating in the inferential evidentiality task. The remaining 12 missing data points were a result of participants not passing the control questions for the FBU test(s). There were again no participants who failed all four control questions. Another important point to mention here is the strikingly low mean of inferential evidentiality scores. This can be explained by the optional nature of evidentiality structures in the English language. Direct evidentiality scores were not included in this table or in any of the analyses given that there was only one occurrence of direct evidentiality use, presenting insufficient variability. More detail on evidentiality performance is provided in the next section.

**TABLE 5:** Descriptive statistics for the English dataset.

	Mean	SD	Range	Max. score	Missing data points (*N* and %)
Short‐term memory	12.5	4.01	0–22	36	0
Receptive vocabulary	58.82	14.91	11–77	100	0
Source monitoring	60.82	29.95	0–100	100%	0
False belief understanding	2.14	1.53	0–5	5	12 of 250 (4.8%)
Evidentiality (inferential)	3.41	10.55	0–60	100%	2 of 50 (4%)

Table 6 demonstrates the correlations between the continuous predictor and control variables using Spearman's correlation. Contrary to the Turkish dataset, there was no significant relationship between false belief performance and source monitoring. False‐belief performance also did not correlate with evidential marker proficiency, vocabulary performance, or short‐term memory, but it did correlate significantly with age (*r*(48) = .27, *p* < .05). Among the control variables, age and vocabulary were positively correlated with source monitoring performance (*p*s < .01), but short‐term memory was not. Inferential evidentiality performance did not correlate with any of the variables.

**TABLE 6:** Correlations between the predictor and control variables.

Variable	1	2	3	4	5	6
1. Age	1					
2. Vocabulary	.57***	1				
3. Short‐term memory	.40**	.47***	1			
4. Source monitoring	.59***	.53***	.15	1		
5. Evidentiality (inferential)	.14	.13	.11	.19	1	
6. False belief understanding	.27*	.15	.14	.12	.11	1

* *p* < .05

** *p* < .01

*** *p* < .001.

#### Evidentiality performance (exploratory)

Evidentiality performance was an exploratory variable for English‐speaking children to measure their likelihood of using lexical and syntactic means to describe witnessed and non‐witnessed events. For direct evidentiality, there was only a single instance, a complement‐clause construction with a perception verb; “Me saw you wash[ed] the baby”. As a result, there was too little variation to include the results of direct evidentiality performance in any of the analyses. For the inferential evidentiality, English‐speaking children were slightly more likely to mark their sources of evidence, though still much less so than their Turkish‐speaking peers. Ten percent used lexical means to mark inferential evidentiality when talking about a non‐witnessed past event (8% of children specified the source of evidence between 1% and 25% of the times they noticed the change in the toys, and 2% specified it between 26% and 60% of the times), 86% did not use any evidential expressions, and a further 4% did not complete this task. Children who specified their sources of evidence all used the mental verb “think”, such as “I think the plate is broken”.

#### Predictors of source monitoring performance

Multiple linear regression was conducted to analyze the predictors of English‐speaking children's source monitoring performance, following the same approach as with the Turkish dataset. The final model with the significant contributors is presented in Table 7.

**TABLE 7:** Multiple linear regression model for source monitoring performance in the English dataset.

Residuals				
Min	1Q	Median	3Q	Max
−49.951	−16.114	−0.774	16.353	44.912

Coefficients				
	Estimate	SE	*t* value	Pr(>|*t*|)
(Intercept)	−107.6120	29.7494	−3.617	.000737***
Age	2.2138	0.6762	3.274	.002019**
Gender (Girls)	14.3683	6.4288	2.235	.030316*
Vocabulary	0.6248	0.2431	2.570	.013476*

*Note*: Significance codes: ‘***’ .001, ‘**’ .01 ‘*’ .05.

Age was the most significant predictor of source monitoring performance, followed by receptive vocabulary performance, and being a girl. As in the model for Turkish, receptive vocabulary was a significant predictor of source monitoring, but contrary to the Turkish model, FBU was not a predictor of English‐speaking children's source monitoring abilities.

#### Predictors of false belief performance

Generalized linear mixed‐effects models were used to analyze the factors that impact English‐speaking children's false‐belief performance. All fixed and random variables were included in the initial model, except for two differences from the Turkish dataset. The English dataset was collected by two experimenters; therefore, experimenter was included as a random factor. Additionally, as explained previously, direct evidentiality measurement resulted in only one case for English‐speaking children and was excluded from the analyses. The same backward selection principle as before was used. The analysis demonstrated that none of the variables included in the model significantly contributed to explaining the FBU performance of English‐speaking participants.

### Results from the combined dataset

In this section, the results from the Turkish and English datasets were combined, to investigate hypotheses 3 and 4 regarding the effects of speaking Turkish on children's overall FBU performance. To achieve this, the Turkish and English datasets were merged, and language was added as a variable. Following this, a generalized linear mixed‐effects model was fitted to evaluate the predictors of FBU performance. Similar to the previous analyses, the principle of backward selection was followed for this analysis. It is important to note that the initial model included all the fixed and random variables except for the two evidentiality proficiency variables, as these tasks were not comparable across languages (see the Methods section for detailed explanation). Instead, we included an interaction between language and source monitoring in the model to investigate the role of source monitoring as a bridge between evidentiality and FBU. Table 8 demonstrates the final model.

**TABLE 8:** Generalized linear mixed‐effects model for the false belief understanding performance in the combined dataset.

Random effects			
Groups	Name	Variance	SD
ID	(Intercept)	.9887	.9943
Item	(Intercept)	.2564	.5064

Fixed effects				
	Estimate	SE	*z* value	Pr(>|*z*|)
(Intercept)	−0.71304	0.34833	−2.047	.04066*
Language (Turkish)	0.64464	0.30377	2.122	.03383*
Gender (girls)	0.55304	0.31240	1.770	.07668.
Short‐term memory	0.28243	0.16532	1.708	.08757.
Source monitoring	−0.03164	0.21378	−0.148	.88235^†^
Language (Turkish) × source monitoring	0.84694	0.31059	2.727	.00639**

*Note*: Number of observations: 475, groups: ID, 99; Item, 5. Significance codes: ‘**’ .01, ‘*’ .05, ‘.’ .1, ‘^†^’ 1.

This overall look at the combined dataset revealed that speaking Turkish predicted better FBU performance. Additionally, a significant interaction between language and source monitoring performance indicates that for Turkish‐speaking children, but not for English‐speaking children, better source monitoring skills were associated with better FBU performance.

## DISCUSSION

Our aim with this cross‐linguistic study was to investigate the impact of evidentiality on 3‐and 4‐year‐old Turkish‐speaking children's source monitoring skills as well as the impact of evidentiality production, source monitoring skills, and language (Turkish vs. English) on FBU. The results revealed that (1) As hypothesized, Turkish‐speaking children's evidentiality performance (direct evidentiality only) predicted their source monitoring performance, alongside their vocabulary and FBU performances, (2) As expected, Turkish‐speaking children's source monitoring performance predicted their FBU, alongside age and gender (being a girl); whereas contrary to our hypothesis, source monitoring was not a predictor for English‐speaking children's FBU, (3) In the crosslinguistic dataset, as hypothesized, speaking Turkish predicted better FBU, and (4) An interaction between language and source monitoring indicated that source monitoring predicted FBU only for Turkish‐speaking children.

### Source monitoring and evidentiality: Thinking and communicating about sources of information

First, let us consider the predictors of source monitoring performance in Turkish‐speaking children. As hypothesized, evidentiality performance predicted source monitoring performance. Given that the function of evidential markers is to mark the sources of knowledge linguistically, being able to use evidentiality accurately requires us to track back in our memory, remember how we gathered the information, and choose the appropriate marker based on this. The finding that evidentiality predicts source monitoring in Turkish‐speaking 3‐ and 4‐year‐olds, supports the proposal by Aksu‐Koç et al. (2009) that exposure to and production of obligatory grammatical evidentiality equips the user with this almost automatic ability to track back the origins of their information.

It should be noted, though, that only one of our evidentiality measures, direct, but not inferential, evidentiality performance, predicted source monitoring performance. This finding might be related to direct evidentiality being acquired earlier in development (e.g., Aksu‐Koç et al., 2009), which means that direct evidentiality is likely the evidentiality structure that is more robustly used in this age group. Additionally, the difference between the structures of the evidentiality tasks might have played a role. The direct evidentiality task required children to retell a sequence of events they witnessed in a story structure, whereas the inferential task required them to respond to a set of individual changes to toys in a conversational structure, and the difference between a coherent story structure and a dynamic conversation may have affected children's use of evidentials.

FBU was the strongest predictor of source monitoring for Turkish‐speaking children, which aligns with previous research that suggests a relationship between FBU and source monitoring (e.g., Welch‐Ross, 2000). Importantly, better FBU performance also predicted better source monitoring skills, which implies a possible bi‐directional relationship between these two skills.

Receptive vocabulary skills also predicted source monitoring performance, and given the verbal demands of the task, this is an expected outcome. For English‐speaking children, however, the predictors of source monitoring were different. While receptive vocabulary predicted source monitoring in both languages, for English‐speaking children being a girl and being older were other predictors, and FBU did not play a role in better source monitoring skills. This difference suggests that there might be different pathways to source monitoring success in different languages.

### False belief: Relations with language and source monitoring

We now turn to the predictors of FBU performance. Given that understanding our own and others' false beliefs also requires tracking and then updating our knowledge, an expectation that there would be a relation between evidentiality and FBU can be justified. Additionally, previous research suggested that Turkish children's better FBU performance might be the result of the characteristics of the Turkish language, specifically the existence of evidential markers (Lucas et al., 2013). We suggested that such a connection can be explained in a stepwise way: evidentiality proficiency predicting source monitoring, and source monitoring predicting FBU. Indeed, in the current study, we did not observe a direct impact of evidentiality performance on FBU. Instead, we observed an indirect impact where direct evidentiality predicted better source monitoring performance, which in turn predicted better FBU performance for Turkish‐speaking children. Based on the Thinking for Speaking tradition (e.g., Slobin, 1996), Matsui and Fitneva (2009) discuss the possibility that, for children speaking a language where there is constant use of evidentiality, there might be an impact on their cognitive abilities compared to children who do not automatically check the sources of their information as a function of their language.

When looking at the combined results and hypothesis 3, as expected, speaking Turkish did yield better FBU performance. Further, a significant interaction between language and source monitoring suggests that source monitoring skills were only related to FBU in Turkish‐speaking children. This finding partially confirmed Lucas et al.'s (2013) suggestion. While we expect Turkish evidentiality to play a central role in the crosslinguistic differences, the crosslinguistic analysis did not test the impact of evidentiality directly, because, as explained in the methodology section, it is not straightforward to compare Turkish‐ and English‐speaking children's evidentiality performance. The comparison of evidentiality was challenging given that both direct and inferential evidentiality were scarcely used by English‐speaking children. Only five out of 50 participants expressed the source of their knowledge in the inferential evidentiality task, and only a single participant marked source of knowledge in the direct evidentiality task.

As a way to combat this challenge with comparing evidentiality performance in two languages, including language as an encapsulating variable confirmed that there was indeed an impact of language that determined the difference in FBU performance. Especially when the interaction between language and source monitoring was evaluated, it was highlighted that Turkish‐speaking children's FBU success could be attributed to their language and source monitoring skills. This outcome once again provides us with a somewhat indirect relation where Turkish‐speaking children's direct evidentiality skills predicted their better source monitoring skills, which in turn predicted their better FBU. In other words, we can argue that speaking a language with obligatory evidentiality supports children's source monitoring skills by pushing them toward monitoring the sources of their information, and this constant monitoring supports their ability to track their own and others' mental states.

Focusing on the predictors of FBU for English‐speaking children, surprisingly, source monitoring abilities did not predict FBU performance, neither were these two correlated. At this point, it is important to highlight that none of the variables significantly predicted English‐speaking children's FBU performance, including age, which has been repeatedly found to predict mental state reasoning (see Wellman et al., 2001 for a review). One of the reasons for this result might be the relatively low success rate in English‐speaking children's FBU (*M* = 2.14) which resulted in non‐significant correlations between FBU and other predictor variables.

For Turkish‐speaking children, while age and source monitoring performance were expected to predict FBU performance, finding such a strong impact of gender for Turkish‐speaking children was surprising. However, there are indeed suggestions in the literature that girls may have an advantage in mental state reasoning. For instance, Selçuk et al. (2018) used Wellman and Liu's (2004) ToM scale to measure 34‐ to 80‐month‐old Turkish children's ToM performance. Their results also revealed that girls outperformed boys, though gender did not predict ToM performance in the regression analysis. Similarly, a post‐hoc analysis of two large sample FBU studies on British children between the ages of 2 and 6 years demonstrated an advantage for girls (Charman et al., 2002). Discussions about gender advantage include issues such as differences in familial interactions where parents were found to discuss emotions and mental states with girls more than with boys (e.g., Leaper et al., 1998). It would be far‐reaching to make any assumptions about Turkish girls' family interactions in the context of the current study, but this would be an interesting area to address in future research.

### Future directions

The findings of this study align with the previous suggestion that being a Turkish‐speaking child contributes to better mental state reasoning as measured by tests of FBU, when compared to English‐speaking children. However, the results of this study still leave some questions open regarding the role of evidentiality in the relation between language and FBU. For the crosslinguistic comparison, the evidentiality tasks we used only elicited limited use of evidentiality in the English‐speaking children. This is in itself an important finding, as this confirms that a language with obligatory grammatical marking of evidentiality indeed stimulates children to communicate spontaneously about their sources of information much more frequently than a language with optional lexical marking. While the tests we used thus provided valid measures of spontaneous use of evidential marking, they also made it difficult to evaluate English‐speaking children's proficiency with evidential marking, as the tasks did not put pressure on children to mark their sources of information. Additionally, due to the differences in structure between the direct and inferential evidentiality tasks we used (story vs. conversation), it is difficult to evaluate the finding that only direct, not inferential, evidentiality predicted FBU in Turkish‐speaking children. To combat these issues, future research should use an innovative evidentiality production measurement that can compare Turkish and English speakers' performances in both evidentiality structures. This can be achieved by creating a task where speakers of both languages are motivated to declare the sources of their knowledge beyond the linguistic obligation.

## CONCLUSIONS

Explicit FBU has strong ties with language proficiency and other cognitive processes. In this cross‐linguistic research, we aimed to enhance our understanding of the universality or language dependency of this socio‐cognitive ability. Specifically, we focused on evidentiality to address the linguistic differences between Turkish and English, and source monitoring as the cognitive skill that might support FBU. This study revealed that direct evidentiality performance impacted Turkish‐speaking children's source monitoring, and that their source monitoring performance in turn predicted their FBU. Examining predictors of FBU in the cross‐linguistic sample, we found a main effect of language indicating a significant advantage for Turkish‐speaking children as well as an interaction between language and source monitoring, indicating that source monitoring only predicted FBU for Turkish‐speaking children. The current study is an important step toward disentangling specific linguistic and cognitive abilities that contribute to children's ability to comprehend and reflect on their own and others' false beliefs.

## Data Availability

Data, code, materials, and the preregistration for this research are available at the following URL: https://osf.io/mks6h/.
